# High resolution X-ray spectra of the time evolution of emission from metastable electronic states of highly charged Ni-like ions

**DOI:** 10.1140/epjd/s10053-024-00872-0

**Published:** 2024-06-25

**Authors:** Timothy Burke, Endre Takacs, Dipti, Adam Hosier, Galen O’Neil, Joseph Tan, Hunter Staiger, Aung Naing, Joan Marler, Yuri Ralchenko

**Affiliations:** 1 Department of Physics and Astronomy, Clemson University, Clemson, SC 29634 USA; 2 National Institute of Standards and Technology, Gaithersburg, MD 20899 USA; 3 International Atomic Energy Agency, 1400 Vienna, Austria; 4 National Institute of Standards and Technology, Boulder, CO 80305 USA

## Abstract

**Abstract:**

Metastable levels of highly charged ions that can only decay via highly forbidden transitions can have a significant effect on the properties of high temperature plasmas. For example, the highly forbidden 3d$^{10}$ $_{J=0}$ - 3d$^9$4 s $(\frac{5}{2},\frac{1}{2})_{J=3}$ magnetic octupole (M3) transition in nickel-like ions can result in a large metastable population of its upper level which can then be ionized by electrons of energies below the ground state ionization potential. We present a method to study metastable electronic states in highly charged ions that decay by x-ray emission in electron beam ion traps (EBIT). The time evolution of the emission intensity can be used to study the parameters of ionization balance dynamics and the lifetime of metastable states. The temporal and energy resolution of a new transition-edge sensor microcalorimeter array enables these studies at the National Institute of Standards and Technology EBIT.

**Graphical abstract:**

NOMAD calculated time evolution of the ratio of the Ni-like and Co-like lines in Nd at varying electron densities compared with measured ratios
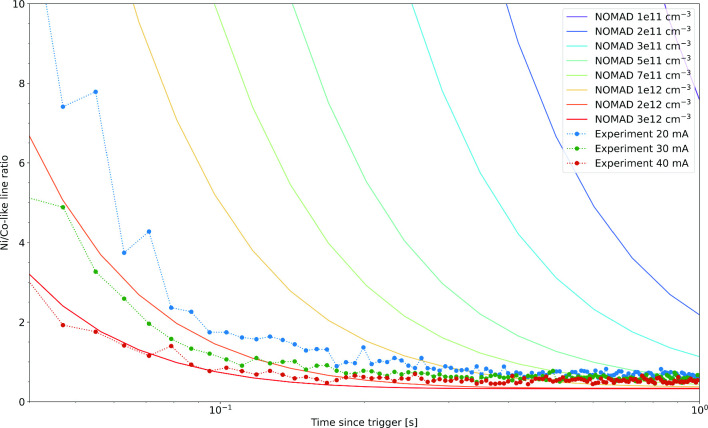

## Introduction

The steady-state charge state distribution of high temperature plasma is the result of a balance of all electron exchange processes. In plasmas containing highly charged ions, this balance is the result not only of the charge states present but also of the electronic state population distributions within each charge state. Emission from impact excited ions give rise to characteristic spectra that can be used to study the parameters of the plasma as well as give insight into the atomic properties of the ions [1].

Electron beam ion traps (EBITs) contain non-Max wellian highly charged ion plasmas consisting of highly charged ions in a range of charge states and the monoenergetic electron beam used to create them. By electrostatically accelerating electrons and then compressing them with a strong magnetic field, a high density electron beam is formed which impact ionizes neutral atoms injected into the system either through ballistic gas injection or, e.g., in the case of solids, singly charged ions from a Metal Vapor Vacuum Arc (MeVVA) external ion source. Ions in the trap are attracted radially to the space charge of the electron beam and confined axially by electrostatic elements called drift tubes. The ions undergo further impact ionizations before reaching a steady-state charge state distribution.

The ionization balance as well as the excited state populations of each ion are a function of electron beam energy, density, and cook time (time spent interacting with the electron beam). The monoenergetic nature of the electron beam allows charge state selectivity and by collecting time-dependent spectra at various densities and energies, we can study the dynamics of atomic processes occurring in high temperature plasmas.

Significant to the ionization balance involving ions with a low-lying metastable state are electron impact excitations, which can lead to radiative cascade decay into the metastable state. Since these states can have sufficiently long lifetimes, further electron impacts can ionize them to higher charge states, a process often referred to as ladder ionization. It has been found significant in few-electron charge states outside of a closed electronic shell, for example Pd-like ions [2–5], Ag-like ions [6, 7], and Ni-like ions  [8–12]. It is particularly important in non-Maxwellian, quasi-monoenergetic plasmas with electron energies below the ionization threshold as higher charge states would not be present without this ionization from an excited state. Furthermore, these metastable decays are interesting from a theoretical perspective as a benchmark of QED calculations; measurements of these highly forbidden transitions provide data on otherwise rare events [13, 14].

**Fig. 1:**
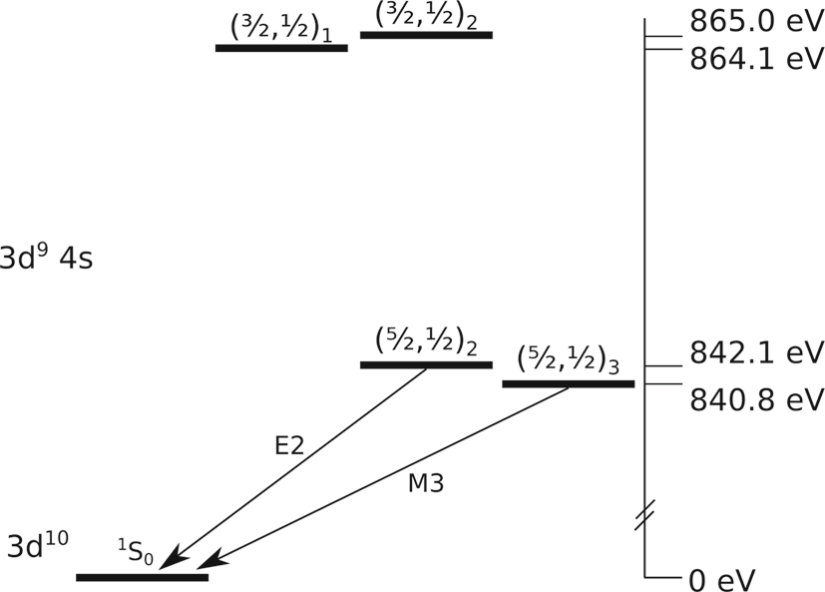
The electronic configuration of the first excited states of Ni-like Nd. The relevant forbidden transitions are labeled. Energies are from [17]. Ni-like Pr has a similar structure but with energies $\approx 45$ eV lower

We particularly investigate the metastable first excited state in Ni-like ions in this work. Figure 1 shows the metastable 3d$^9$4 s $(\frac{5}{2},\frac{1}{2})_{J=3}$ state in Ni-like Nd. To first approximation, the only significant depopulation branches are the magnetic octupole (M3) transition to ground ($\tau \approx$ 2.5 ms), excitation to higher excited states, or ionization to the Co-like charge state. This statement is true if the hyperfine interaction can be neglected as in zero nuclear spin isotopes, otherwise, hyperfine effects might affect the metastability of the level [11, 15]. For metastable Ni-like ions, the hyperfine interaction can lead to mixing of the 3d$^9$4 s $(\frac{5}{2},\frac{1}{2})_{J=3}$ and 3d$^9$4 s $(\frac{5}{2},\frac{1}{2})_{J=2}$ states, thus opening an E2 decay channel for the radiative decay of the metastable state. This mixing reduces the population of the affected magnetic sublevels of the metastable state and is known as hyperfine quenching [16].

In the case of natural Nd, the isotopic abundance is $\approx$80% even nucleon isotopes with the other 20% consisting of $^{143}$Nd and $^{145}$Nd, both with nuclear spin $I=7/2$, while the natural abundance of Pr is 100% $^{141}$Pr with nuclear spin $I=5/2$. Comparing spectra for these Ni-like ions, the effects of hyperfine quenching can be potentially observed. In this paper we report our approach to investigate this effect via recording the time-dependent evolution of Ni-like and Co-like spectral lines acquired with high x-ray energy and time resolution. The transition-edge sensor (TES) spectrometer on the NIST electron beam ion trap operating in fast-switching mode allowed these studies.

**Fig. 2:**
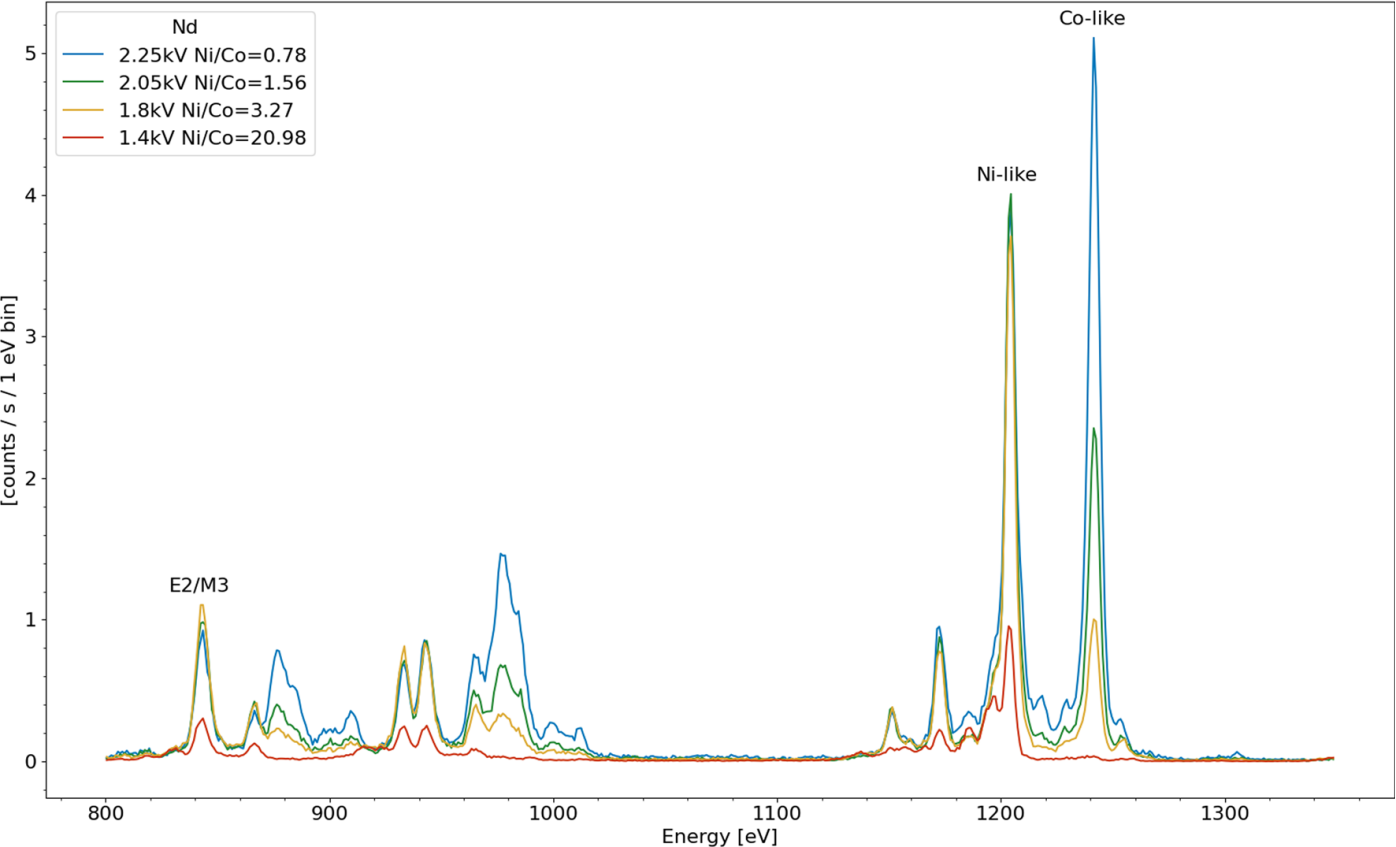
Integrated x-ray spectra taken at four electron beam energies demonstrating the strong dependence of line intensity as a function of electron beam energy. The legend indicates the electron beam energy and also includes the ratio of the integrated intensity under the peak between the strongest Ni-like 3d$^{10}$–3d$^9$4f (1203 eV) and strongest Co-like 3d$^{9}$–3d$^8$4f (1241 eV) spectral features

## Experiment

The method described here requires fast observation of x-ray spectra of the plasma as a function of time triggered with turning on the electron beam. Singly charged ions are injected into the trap by the MeVVA, the trap potentials are raised, then the electron beam is switched on and the evolution of the charge state balance to steady state is observed by x-ray signature. After some time, when steady-state population is reached, the electron beam is turned off and the trap is dumped, this cycle is repeated many times for the measurement.

The buildup of intensity of each line as they reach steady-state emission rates after switching the electron beam back on can provide information about the processes affecting their upper level population of the transitions (e.g., electron impact and charge exchange cross sections, lifetimes, and trap losses). Comparison with theory and systematic changes of the electron beam and trap parameters can selectively provide experimental results for the processes in the trap. In principle the evolution of the charge state balance after removing the electron beam can also be observed with appropriate timing and sufficient collection statistics. The observation of the long lifetime decay fluorescence after the electron beam is removed provides a direct measurement of lifetime [11]. Our time resolution and detection efficiency allows for such measurements, but that analysis is not part of this report.

We developed this technique with x-rays for measurements on the NIST EBIT described in  [18]. The electrostatic acceleration of the electron beam is achieved by the voltages between the cathode, anode, and drift tube assemblies. By quickly dropping the anode voltage of the electron-gun, the fast and complete suppression of the electron beam can be achieved. We make use of a DEI PVX-4130 high voltage pulse generator to achieve anode switching on the order of 100 ns.

Spectra were collected with the NIST transition-edge sensor (TES) microcalorimeter [19]. This allows us to achieve $\mu$s resolved spectra with approximately 4 eV photon energy resolution over 500 eV–10,000 eV x-ray energy range. The time resolved spectra are important in the measurement of the initial charge state buildup and for x-ray fluorescence studies.

By varying the electron beam energy and density, the effects on the charge state evolution and steady-state balance are studied. The population of the metastable state is highly sensitive to the electron beam density since, for increasing densities, impact excitation becomes the dominate depopulating process of the Ni-like 3d$^9$4 s $(\frac{5}{2},\frac{1}{2})_{J=3}$ metastable level  [20]. This allows for the tuning and fitting of these parameters in theoretical collisional radiative models and by extension, potentially determining relative values for the electron impact cross sections.

## Results

Figure 2 shows x-ray spectra taken with four different electron beam energies. The spectra are labeled with the applied accelerating potentials, which are within a few percent of the true electron beam energy, which is affected by the space charge potential of the electron beam itself. The ratio of the populations of Ni-like ions to Co-like ions present in the trap can be estimated from the ratio of the intensities of the two strongest (and well isolated) spectral features for those two species. Used to calculate the ratio are Ni-like feature (3d$^{10}$–3d$^9$4f) at 1203 eV and the Co-like feature (3d$^{9}$–3d$^8$4f) at 1241 eV, both of them dominated by strong electric dipole allowed (E1) transitions. The ratios of the intensities are listed in the legend and decrease rapidly (from $\approx$ 21 to almost zero) as the electron beam energy is varied from far below the ground state Ni-like ionization energy (2134 eV [21]) to above that value at $\approx$2250 eV.

However, notably, the Co-like feature is still present far below the ionization energy (e.g., in the 1.8 kV electron beam spectra), the presence of the Co-like ions is due to ionization of Ni-like ions which have been excited to the metastable state which occurs as long as the electron beam energy is above $\approx 1300$ eV. At the highest, $\approx$2250 eV, electron beam energy direct ionization from the ground state sets in and the ionization balance begins to favor the higher ionized Co-like ions.

**Fig. 3:**
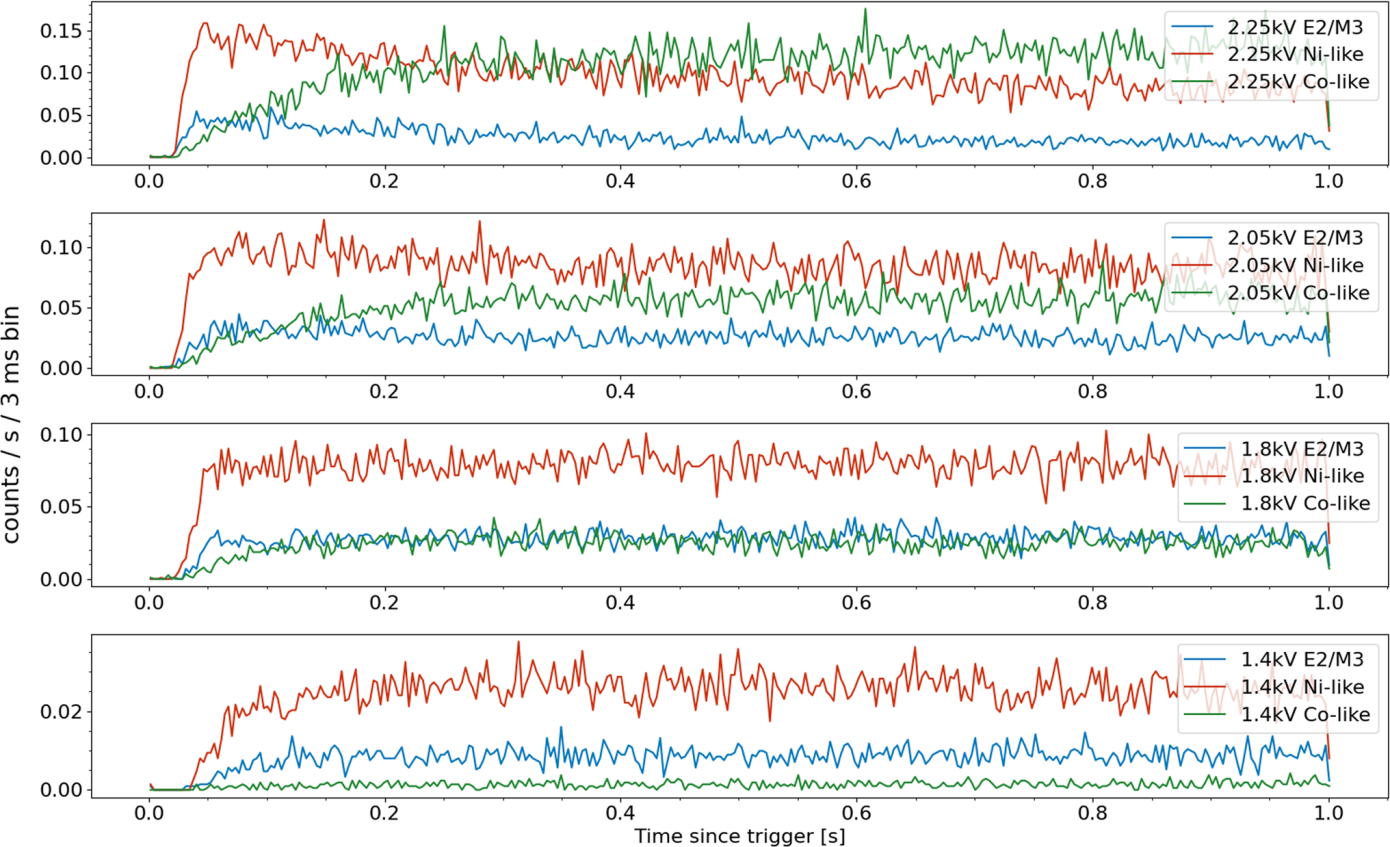
Time evolution of the strongest Ni-like (3d$^{10}$–3d$^9$4f) and Co-like (3d$^{9}$–3d$^8$4f) features in Nd as well as that of the E2/M3 blend for Ni-like ions at four electron beam energies. Ground state direct ionization of Ni-like ions can only occur in the top panel where the electron beam has energy greater than 2134 eV

The time-dependent evolution of these strongest features of Ni-like and Co-like Nd lines, as well as the E2/M3 blend (at 840 eV), are shown in Fig. 3. The separation of the E2 and E3 lines are smaller than our current experimental x-ray energy resolution, therefore the E2/M3 blend includes both the long-lifetime M3 and the about six orders of magnitude faster E2 transitions [20]. Due to experimental considerations, the beam currents must decrease with lower accelerating potential in order to mitigate sputtering caused by the decrease in electrostatic focusing provided by higher potentials.

From these time-dependent traces, one can see the fluorescence from the Ni-like E2/M3 feature rises faster before the appearance of the Co-like strong transition. Again even when the electron beam energy is around 2 keV (below the ground state ionization from Ni-like to Co-like) the Co-like line intensity grows to a steady-state value from continuous creation of these ions. In all four panels, the Co-feature has longer time constants than the Ni-like fluorescence.

**Fig. 4:**
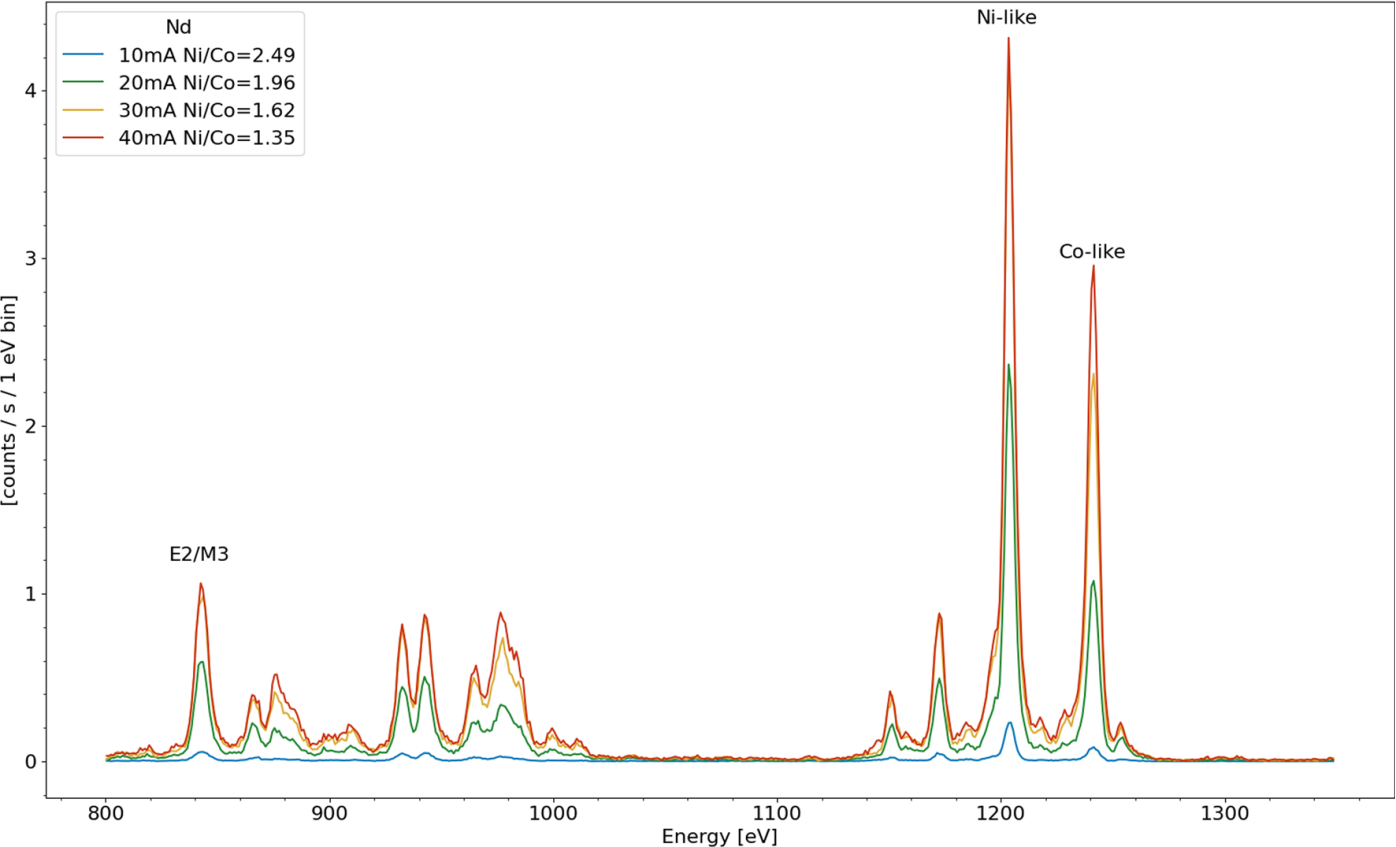
Nd spectra taken at five electron beam currents with electron beam energy fixed at 2150 eV demonstrating the line ratio dependence by the electron density. The legend includes the intensity ratio between the strongest Ni-like 3d$^{10}$–3d$^9$4f (1203 eV) and strongest Co-like 3d$^{9}$–3d$^8$4f (1241 eV) features

**Fig. 5:**
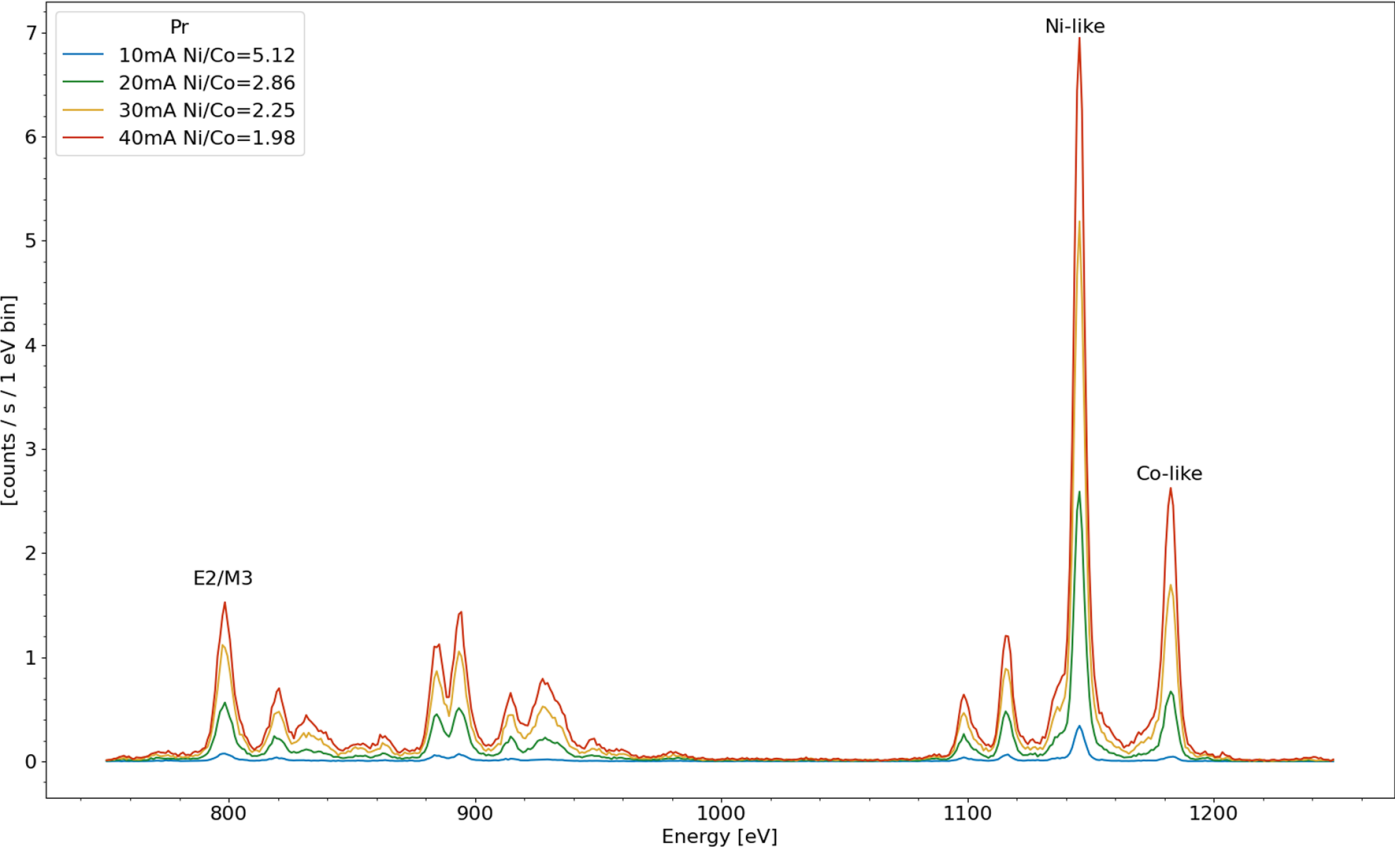
Pr spectra taken at five electron beam currents at constant energy (2050 eV) demonstrating the line ratio dependence. The legend also includes the intensity ratio between Ni-like 3d$^{10}$– 3d$^9$4f (1142 eV) and strongest Co-like 3d$^{9}$–3d$^8$4f (1182 eV) features

**Fig. 6:**
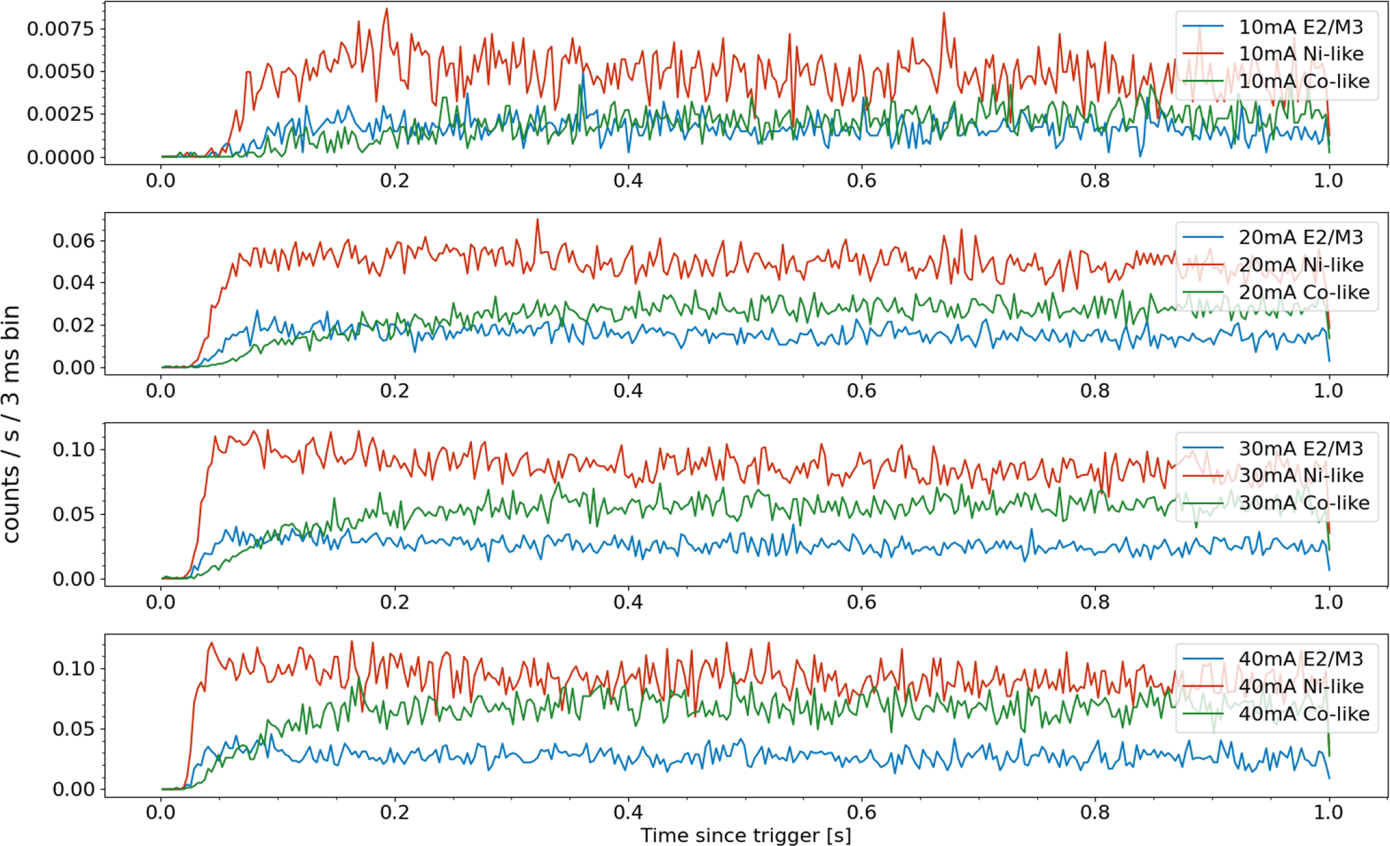
Time evolution of the strongest Ni-like and Co-like in Nd lines as well at the E2/M3 blend for different electron beam currents at a constant 2150 eV beam energy

**Fig. 7:**
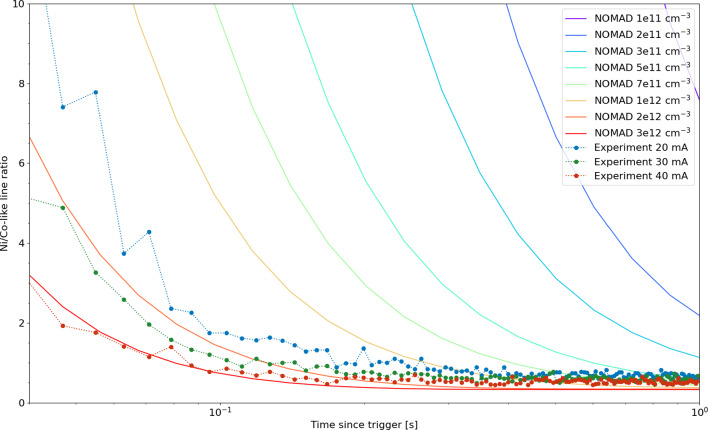
NOMAD calculated time evolution of the ratio of the Ni-like and Co-like lines in Nd at varying electron densities compared with measured ratios

Shown in Figs. 4 and 5 are integrated spectra of Nd and Pr, respectively, obtained by varying the beam current, this equates to varying the electron density proportionally assuming that the electron beam radius is the same at different beam currents. The effect of the hyperfine quenching in Pr is apparent when comparing the Ni/Co-like line ratios to those of Nd. Since both of these data sets are taken at energies below the ground state Ni-like ionization energy for their respective ions, the only process to populate the Co-like charge state is ionization from the metastable 3d$^9$4 s $(\frac{5}{2},\frac{1}{2})_{J=3}$ level. Without the hyperfine effect, we would expect Pr to have a higher proportional population of Co-like ions as the Z atomic number dependent M3 lifetime is predicted to be longer (3.3 ms vs 2.5 ms in Nd [22]) and therefore the metastable population is qualitatively expected to be larger. However, this is not the case, Pr has a lower proportion of Co-like ions due to the lower metastable population as a result of the hyperfine induced mixing of the metastable state with its neighboring, E2 allowed, see Fig. 1, state [11, 20].

Finally, in Fig. 6 we see the effect of electron density on the time evolution of the charge states. Collisional-radiative model calculations using the NOMAD package  [23], the non-Maxwellian electron energy distribution function (EEDF) was modeled as a Gaussian with the full width at half maximum of 40 eV. The calculated collisional cross sections were then integrated with this EEDF to generate the collisional rates. This model is compared in Fig. 7 with experimentally measured time dependent line ratios. This figure demonstrates that the Ni/Co-like line ratios as a function of the elapsed time after the injection of the ions into the trap can be a strong indication of electron density. The experimental ratios are multiplied by a constant factor that represents the average A coefficient ratios for the two spectral features other trapping an electron impact dependent parameters. Full scale analyses that include calculations of these parameters can turn the time-dependent measurement into a powerful diagnostic for the otherwise difficult to determine electron density.

## Conclusion

We have used the TES microcalorimeter array on the NIST EBIT for producing time-dependent spectra with high photon-energy and photon-arrival-time resolution. We have also demonstrated our ability to switch the electron beam on and off quickly to take advantage of our advanced x-ray detection features. We took spectra of Nd and Pr at a variety of electron beam parameters and charge state populations. We show, empirically, the electron density dependence of the Ni-like metastable level population and its effect on the charge state distribution of ions in the EBIT.

By comparing the spectral features of Nd and Pr, we qualitatively point out the effect of hyperfine quenching of the metastable state in Ni-like ions. We show this on sample spectra for the two elements under different electron beam conditions by looking at the time evolution of different charge states in the trap one with hyperfine mixing one without.

These measurements provide an extensive data set for theoretical models as well as plasma diagnostics. Further investigation will be conducted in order to quantify the atomic parameters that can be precisely determined from spectra. The long-term goal of the project is to determine electron impact cross sections and metastable lifetimes though fitting with theoretical collisional radiative models.

## Data Availability

This manuscript has no associated data or the data will not be deposited. Data used in this work will not be made available at this time as the data are part of ongoing research.
